# Accelerated pace of frailty in patients with schizophrenia

**DOI:** 10.1016/j.jnha.2024.100412

**Published:** 2024-11-29

**Authors:** Shun Yao, Lijun Wang, Zhiying Yang, Yichong Xu, Xiaoqing Zhang, Yuan Shi, Donghong Cui

**Affiliations:** a Shanghai Mental Health Center, Shanghai Jiao Tong University School of Medicine, Shanghai, China; b Shanghai Key Laboratory of Psychotic Disorders, Shanghai Jiao Tong University School of Medicine, Shanghai, China

**Keywords:** Schizophrenia, Frailty, FI-Lab, PANSS

## Abstract

**Background:**

Schizophrenia is associated with an increased risk of mortality and physical comorbidities, indicating a potentially accelerated frailty process in affected individuals. This study aimed to test association between schizophrenia and frailty using the frailty index based on laboratory markers (FI-Lab).

**Methods:**

A total of 600 patients with schizophrenia and 518 healthy controls, aged between 20 and 69 years were included in the present study. Frailty was assessed using the FI-Lab, incorporating routine laboratory markers, body mass index, and blood pressure measurements. FI-Lab for patients with schizophrenia and healthy controls was compared, with stratification by age group and sex. In addition, robust was defined as FI-Lab ≤ 0.12, pre-frail as 0.12–0.25, and frail as >0.25. Multiple linear regression analysis was used to test the association between schizophrenia and FI-Lab. Multinomial logistic regression was used to test the association between schizophrenia and frailty status. Spearman correlation analysis was performed to assess the relationship between the Positive and Negative Syndrome Scale (PANSS) scores and FI-Lab in schizophrenia patients.

**Results:**

Schizophrenia patients exhibited significantly higher FI-Lab than healthy controls across all age groups, indicating accelerated pace of frailty in schizophrenia patients. Schizophrenia was significantly associated with FI-Lab (β = 0.044, p = 0.004) in the adjusted model. Schizophrenia was significantly associated with both pre-frail status (OR = 2.26, 95% CI = 1.40−3.68, p = 0.001) and frail status (OR = 10.33, 95% CI = 5.65−19.93, p = 0.007) compared to robust status in the adjusted model. Additionally, a positive correlation between FI-Lab and PANSS scores suggests that more severe schizophrenia symptoms correlate with higher degree of frailty.

**Conclusion:**

These findings suggest that schizophrenia contributes to an increased risk of frailty. The FI-Lab provides a quantitative measure of frailty. This underscores the importance of integrating frailty considerations into the treatment and management of schizophrenia.

## 1 Introduction

Frailty is defined as “a condition in which an individual is in a vulnerable state at increased risk of adverse health outcomes and/or dying when exposed to a stressor” [1,2]. Schizophrenia is a severe psychiatric disorder characterized by positive symptoms, negative symptoms and cognitive impairment [3]. Individuals diagnosed with schizophrenia have a mortality risk 2–3 times higher than the general population and experience a reduction in life expectancy of 15–20 years [4]. This excess mortality is largely due to various physical comorbidities. Studies have shown that individuals with schizophrenia are at an increased risk of developing various aging-related diseases, including metabolic disorders, respiratory diseases, cardiovascular diseases, and Alzheimer's disease [[5], [6], [7], [8]]. These observations suggest that schizophrenia may be accompanied by accelerated frailty [9].

Frailty is usually assessed in older adults. Studies have reported that the prevalence of frailty is higher in older patients with schizophrenia compared to the general population [[10], [11], [12]]. Additional data are needed to determine whether patients with schizophrenia experience accelerated frailty from an early stage of life. The accelerated frailty observed in individuals with schizophrenia could serve as a concrete indicator of mortality and physical comorbidities in these patients, providing valuable insights for clinical management and intervention strategies. These insights could help enhance the quality of life and health outcomes for individuals with schizophrenia.

The Rockwood's model of frailty proposes that variability and longitudinal trajectories in health outcomes among people with the same chronological age is strongly related to the extent to which they have accumulated health deficits during life [13]. That is, the more deficits a person presents, the more that person will be frail and, thus, more vulnerable to poorer outcomes (e.g., mortality, disability) [14]. Quantifying these negative health attributes and measuring their cumulative effect can therefore provide a useful estimate of the individual's risk profile [15,16].

This study aims to explore the accelerated pace of frailty in individuals with schizophrenia by frailty index (FI) in each age group. This model of the FI was constructed by routine laboratory tests, plus the body mass index (BMI) and measures of systolic and diastolic blood pressures [17]. The laboratory deficits of the FI (FI-Lab), which precede clinically evident health deficits, reflect a range of general subclinical deficits that originate at the molecular or cellular level and eventually scale up to visible macroscopic organ dysfunction [18].

## 2 Methods

### 2.1 Study participants

Data were drawn from the multi-modal psychiatric disorder cohort funded by the National Key Research and Development Program of China, which aimed to study severe mental disorders through the integration of genetic, imaging, and clinical features. Patients were recruited from multiple institutions including. All patients were recruited through outpatient clinics and were diagnosed by qualified medical professionals. Healthy controls were recruited from the local community through advertisements, and were screened to exclude individuals with a personal or family history of psychiatric disorders. The cohort collected comprehensive demographic information, clinical assessment scales, physical examination data, blood markers, imaging information, and genetic information. The project was approved by the Ethics Review Committee of the Shanghai Mental Health Center, and all participants provided written informed consent. The inclusion and exclusion criteria for this study were as follows:

For schizophrenia patients: (1) All patients met the diagnostic criteria for schizophrenia, as determined by experienced psychiatrists according to the Diagnostic and Statistical Manual of Mental Disorders, Fourth Edition (DSM-IV). (2) Aged between 20–69 years; (3) with complete information for all components of the FI-Lab. For healthy controls: (1) aged between 20–69 years; (2) with complete information for all components of the FI-Lab; (3) without a history of psychotic disorders. Exclusion criteria for both groups were central nervous system diseases, severe physical diseases, substance abuse or dependence, pregnancy, or lactation and any other psychiatric disorders.

Finally, a total of 1118 individuals (600 schizophrenia patients and 518 healthy controls) were included in the present study.

### 2.2 Construction of FI-Lab

The factors included in the FI are based on the following criteria [19,20]: (1) Must increase with age: The factor should show a trend of increasing prevalence or severity as the individual ages. (2) Must be health-related: The factor should have a direct connection to health status or medical conditions. (3) Must be present in at least 1% of the study population: The factor should not be too rare and should be present in a significant minority of the population being studied. (4) Must not be present in 80% of the study population prior to the age of 80: The factor should not be overly common in younger individuals and should typically manifest or become more prevalent as people age.

We constructed an FI-Lab panel using 34 variables based on 31 routine laboratory blood markers, BMI, and systolic and diastolic blood pressures. As reported in previous studies, all variables were coded according to normal reference intervals: a code of “zero” indicates values within the normal range, while a code of “one” indicates any values outside of the normal reference range (Table S1). For each subject, the FI-Lab score was calculated as the number of deficits divided by the total number of tests. In addition, those with a FI-Lab ≤ 0.12 were defined as robust, those between 0.12 and 0.25 as pre-frail, and those with FI-Lab > 0.25 as frail [21,22].

### 2.3 Assessment of symptoms of schizophrenia

The positive and negative syndrome scale (PANSS) is a well-established, validated clinical tool that measures positive symptoms, negative symptoms, and general psychopathology through a structured interview [23]. Patients were evaluated using the PANSS, which includes 30 items across three subscales: positive symptoms (7 items), negative symptoms (7 items), and general psychopathology (16 items). Each item is rated on a 7-point scale from 1 (absent) to 7 (extreme), reflecting the past week's symptom severity. The assessments were conducted by trained clinicians who had undergone standardized training to ensure reliability and consistency in scoring. The total PANSS score, summing all three subscales, indicates symptom severity, with higher scores denoting greater severity.

### 2.4 Assessment of covariates

Covariates collected included demographics (age, sex, residence status, marital status, education level), smoking habits, weight, and height. BMI was calculated as weight divided by height squared (kg/m^2^). Participants were categorized as current smokers if they reported daily smoking, former smokers if they had smoked continuously for more than six months, or non-smokers. The cognitive function of participants was assessed using the Repeatable Battery for the Assessment of Neuropsychological Status (RBANS) [24].

### 2.5 Statistical analysis

Group differences were assessed using Student’s *t*-test for normally distributed variables and the Mann-Whitney *U* test or Kruskal-Wallis H test for non-normally distributed variables. Results for normally distributed variables are expressed as mean and standard deviation (SD), and as median and interquartile range (IQR) for non-normally distributed variables. The χ² test was used for categorical variables, presenting results as counts and frequencies. Multiple linear regression was used to test the association between schizophrenia and FI-lab. Multinomial logistic regression was used to test the association between schizophrenia and frailty status. Odds ratios (ORs) and 95% confidence intervals (CI) were documented. Two models were developed: a crude (unadjusted) model to assess the direct association of each variable with frailty, and an adjusted model that included schizophrenia, age, sex, marital status, education level, smoking habits, RBANS score, and interaction terms between schizophrenia and age or sex. All variables were entered simultaneously into the model. Spearman correlation analysis was used to explore the relationship between FI-Lab and disease severity (measured by PANSS) in the schizophrenia patients. Correlation coefficients and p-values were calculated, with results presented in scatter plots and fitted curves. Statistical analyses were conducted in R (version 4.3.2, R Foundation for Statistical Computing, Vienna, Austria). Statistical significance was set at a p-value threshold of <0.05 (two-tailed).

## 3 Results

### 3.1 Study population characteristics

A total of 1118 subjects were included in this study, comprising 600 patients with schizophrenia and 518 healthy controls. Demographic and baseline characteristics are detailed in Table 1. The schizophrenia group showed a significantly higher proportion of males, mean age, and smoking rate, but a lower education level compared to the control group. The median of FI-Lab for schizophrenia patients was 0.24 (0.18, 0.26), significantly higher than that of healthy controls at 0.18 (0.12, 0.21). The prevalence of pre-frailty and frailty in patients with schizophrenia was 60.8% and 29.4%, respectively.

**Table 1:** Demographic characteristics of study population.

	Schizophrenia	Control	p-value
	(N = 600)	(N = 518)	
Sex			
Male	341 (56.8%)	166 (32.0%)	<0.001
Female	259 (43.2%)	352 (68.0%)	
Age			
Mean (SD)	45.0 (16.3)	37.6 (13.6)	<0.001
Resident			
Rural	340 (56.7%)	398 (76.8%)	<0.001
Urban	260 (43.3%)	120 (23.2%)	
Marriage			
Unmarried	270 (45.0%)	230 (44.4%)	<0.001
Married	255 (42.5%)	263 (50.8%)	
Divorced	75 (12.5%)	25 (4.8%)	
Education			
Primary school or below	33 (5.5%)	23 (4.4%)	<0.001
Middle or High school	474 (79.0%)	163 (31.5%)	
College or above	93 (15.5%)	332 (64.1%)	
Smoking habit			
Never	428 (71.3%)	459 (88.6%)	<0.001
Former	84 (14.0%)	13 (2.5%)	
Current	88 (14.7%)	46 (8.9%)	
RBANS score			
Mean (SD)	69.1 (17.4)	93.9 (16.1)	<0.001
Frailty index			
Median (Q_1_, Q_3_)	0.24 (0.18, 0.26)	0.18 (0.12, 0.21)	<0.001
Frailty status			
Robust	59 (9.8%)	162 (31.3%)	<0.001
Prefrail	365 (60.8%)	321 (61.9%)	
Frail	176 (29.4%)	35 (6.8%)	

RBANS: Repeatable Battery for the Assessment of Neuropsychological Status; SD: Standard Deviation; Q_1_: 1st Quartile; Q_3_: 3st Quartile.

### 3.2 Comparison of FI-Lab between patients with schizophrenia and healthy control by age

**Fig. 1:**
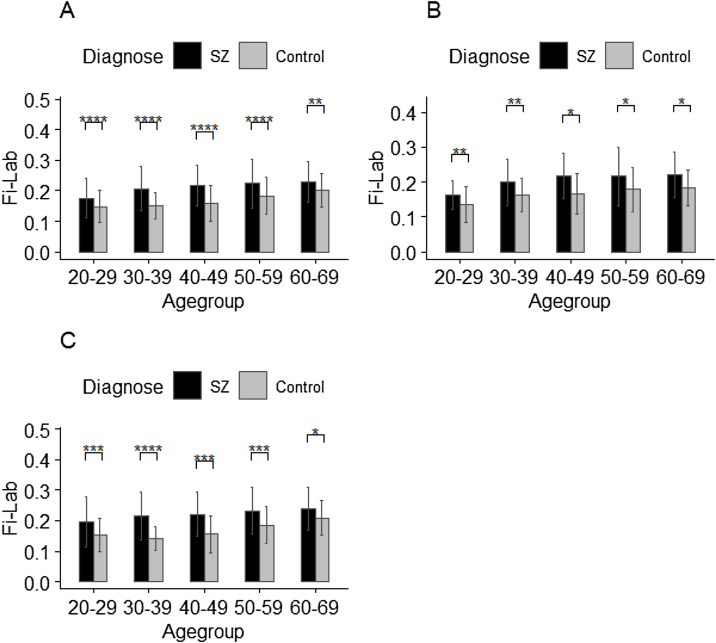
FI-Lab for patients with schizophrenia and healthy controls, stratified by age group and sex. Comparison of FI-Lab between patients with schizophrenia and healthy controls in (A) total participants; (B) males; (C) females. *: p < 0.05; **: p < 0.01; ***: p < 0.001;****: p < 0.0001.

Considering the influence of sex and age on FI-Lab, we stratified subjects into five age groups: 20−29, 30−39, 40−49, 50−59, and 60−69 years. FI-Lab values were compared within each age group between schizophrenia and control groups. Sex-stratified analysis revealed significantly higher FI-Lab values in the schizophrenia group across all age groups for both sex (Fig. 1).

### 3.3 Association between schizophrenia with FI-lab

We analyzed the associations between FI-Lab and potential factors, as presented in Table 2. FI-Lab values were significantly higher in patients with schizophrenia compared to the control group and showed an increase with age. Marital status, education level and smoking habit also had significant effects on FI-Lab. Individuals with poorer cognitive performance (lower RBANS scores) had higher FI-Lab. No significant differences in FI-Lab were observed on sex or resident.

**Table 2:** Comparison of FI-Lab across different groups.

	n (%)	FI-Lab	p-value
Schizophrenia			<.001
No	518 (46.3)	0.18 (0.12, 0.21)	
Yes	600 (53.7)	0.24 (0.18, 0.26)	
Sex			0.684
Male	507 (45.4)	0.18 (0.15, 0.24)	
Female	611 (54.6)	0.18 (0.15, 0.24)	
Age Group			<.001
20−29	366 (32.7)	0.15 (0.12, 0.21)	
30−39	210 (18.8)	0.18 (0.15, 0.23)	
40−49	139 (12.4)	0.18 (0.15, 0.24)	
50−59	207 (18.6)	0.21 (0.16, 0.26)	
60−69	196 (17.5)	0.24 (0.18, 0.26)	
Resident			0.643
Rural	738 (66.0)	0.18 (0.15, 0.24)	
Urban	380 (34.0)	0.18 (0.15, 0.24)	
Marriage			<.001
Unmarried	500 (44.7)	0.18 (0.15, 0.21)	
Married	518 (46.3)	0.18 (0.15, 0.24)	
Divorced	100 (9.0)	0.21 (0.18, 0.26)	
Education			<.001
Primary school or below	56 (5.0)	0.21 (0.18, 0.26)	
Middle or High school	637 (57.0)	0.21 (0.15, 0.24)	
College or above	425 (38.0)	0.18 (0.12, 0.21)	
Smoking habit			0.035
Never	889 (80.4)	0.18 (0.15, 0.24)	
Former	92 (8.3)	0.21 (0.15, 0.26)	
Current	126 (11.34)	0.21 (0.15, 0.24)	
RBANS score			<.001
Quartile 1	288 (25.8)	0.21 (0.18, 0.26)	
Quartile 2	282 (25.2)	0.21 (0.15, 0.26)	
Quartile 3	272 (24.3)	0.18 (0.15, 0.24)	
Quartile4	276 (24.7)	0.18 (0.12, 0.21)	

FI-Lab was presented by median and (Q_1_:1st Quartile, Q_3_: 3st Quartile).

We conducted a multiple linear regression analysis of factors associated with FI-lab (Table 3). In the crude model, schizophrenia (β = 0.050, p < 0.001), being married (β = 0.014, p = 0.002), being divorced (β = 0.038, p < 0.001), higher education (college or above: β = −0.037, p < 0.001), smoking history (former smoker: β = 0.014, p = 0.046; current smoker: β = 0.020, p = 0.015), age (β = 0.002, p < 0.001), and RBANS score (β = −0.001, p < 0.001) were significantly associated with FI-lab. In the adjusted model, schizophrenia remained significantly associated with FI-lab (β = 0.044, p = 0.004), and age also showed a significant positive association (β = 0.001, p < 0.001). Interaction terms between schizophrenia and age or sex were not significant.

**Table 3:** Multiple linear regression analysis of factors associated with frailty status.

	Crude Model		Adjusted Model	
	β (95% CI)	p-value	β (95% CI)	p-value
Schizophrenia				
No	ref		ref	
Yes	0.050 (0.042 to 0.058)	**<.001**	0.044 (0.014 to 0.074)	**0.004**
Sex				
Male	ref		ref	
Female	−0.000 (−0.009 to 0.008)	0.933	0.007 (−0.006 to 0.019)	0.282
Marriage				
Unmarried	ref		ref	
Married	0.014 (0.005 to 0.023)	**0.002**	0.004 (−0.005 to 0.014)	0.331
Divorced	0.038 (0.023 to 0.053)	**<.001**	0.006 (−0.009 to 0.021)	0.435
Education				
Primary school or below	ref		ref	
Middle or High school	−0.002 (−0.021 to 0.017)	0.826	0.004 (−0.014 to 0.023)	0.630
College or above	−0.037 (−0.057 to −0.018)	**<.001**	0.008 (−0.016 to −0.029)	0.399
Smoking habit				
Never	ref		ref	
Former	0.014 (0.000 to 0.027)	**0.046**	0.002 (−0.011 to 0.015)	0.780
Current	0.020 (0.004 to 0.035)	**0.015**	0.003 (−0.012 to 0.017)	0.735
Age	0.002 (0.001 to 0.002)	**<.001**	0.001 (0.001 to 0.002)	**<.001**
RBANS score	−0.001 (−0.99 to −0.001)	**<.001**	−0.001 (−0.000 to −0.001)	0.889
Schizophrenia*Age	–		−0.001 (−0.000 to −0.001)	0.713
Schizophrenia*Sex	–		−0.012 (−0.004 to −0.028)	0.146

Crude Model: Unadjusted model. Adjusted Model: adjusted for schizophrenia, sex, marriage, education, smoking habit, age, RBANS score, interaction term between schizophrenia and age, interaction term between schizophrenia and sex. RBANS: Repeatable Battery for the Assessment of Neuropsychological Status; CI: confidence interval.

### 3.4 Association between schizophrenia with frailty status

Next, we performed a multinomial logistic regression analysis with frailty status as the dependent variable, including the variables that showed significant differences as independent variables (Table 4). In the crude model, schizophrenia, age, being divorced, current smoker, higher education (college or above) and RBANS score were significantly associated with both pre-frail status and frail status (p < 0.05). In the adjusted model, schizophrenia remained significantly associated with both pre-frail status (OR = 2.26, 95% CI = 1.40−3.68, p = 0.001) and frail status (OR = 10.33, 95% CI = 5.65−19.93, p = 0.007) compared to robust status. Age was significantly associated with both pre-frail status (OR = 1.03, 95% CI = 1.01−1.05, p = 0.008) and frail status (OR = 1.07, 95% CI = 1.03–1.11, p < 0.001). The interaction terms between schizophrenia and age or sex were not significantly associated with either pre-frail or frail status, suggesting that the increased risk of frailty in individuals with schizophrenia is independent.

**Table 4:** Multinomial logistic regression analysis of factors associated with frailty status.

	Pre-frail vs. robust				Frail vs. robust			
	Crude Model		Adjusted model		Crude Model		Adjusted model	
	OR(95% CI)	p-value	OR(95% CI)	p-value	OR(95% CI)	p-value	OR(95% CI)	p-value
Schizophrenia								
No	ref		ref		ref		ref	
Yes	3.12 (2.24−4.36)	**<0.001**	2.26 (1.40−3.68)	**0.001**	13.81 (8.63−22.08)	**<0.001**	10.33 (5.65−19.93)	**0.007**
Sex								
Male	ref				ref		ref	
Female	0.64 (0.47−1.04)	0.462	0.93 (0.60−1.43)	0.740	0.87 (0.59−1.29)	0.874	1.46 (0.62−3.49)	0.388
Marriage								
Unmarried	ref				ref			
Married	1.43 (1.05−1.95)	**0.026**	1.00 (0.67−1.50)	0.985	1.57 (1.06−2.34)	**0.026**	0.99 (0.60−1.66)	0.992
Divorced	3.37 (1.63−8.23)	**0.002**	1.80 (0.75−4.31)	0.187	6.32 (2.65−15.08)	**<0.001**	1.65 (0.63−4.33)	0.309
Education								
Primary school or below	ref				ref			
Middle or High school	0.64 (0.25−1.68)	0.366	0.70 (0.25−1.90)	0.477	0.60 (0.21−1.70)	0.334	0.74 (0.24−2.29)	0.601
College or above	0.27 (0.10−0.71)	**0.008**	0.74 (0.26−2.15)	0.585	0.11 (0.04−0.31)	**<0.001**	0.80 (0.23−2.72)	0.717
Smoking habit								
Never	ref				ref			
Former	1.17 (0.71−1.93)	0.540	0.74 (0.42−1.30)	0.297	1.61 (0.88−2.91)	0.118	0.89 (0.45−1.79)	0.752
Current	2.17 (1.05−4.47)	**0.035**	1.14 (0.52−2.48)	0.740	3.52 (1.60−7.73)	**0.002**	1.54 (0.65−3.67)	0.329
Age	1.04 (1.03−1.05)	**<0.001**	1.03 (1.01−1.05)	**0.008**	1.07 (1.06−1.09)	**<0.001**	1.07 (1.03−1.10)	**<0.001**
RBANS score	0.98 (0.97−0.98)	**<0.001**	0.99 (0.98−1.01)	0.229	0.96 (0.95−0.97)	**<0.001**	0.99 (0.98−1.01)	0.649
Schizophrenia*Age	–		1.00 (0.98−1.03)	0.783	–		0.99 (0.96−1.03)	0.693
Schizophrenia*Sex	–		0.70 (0.34−1.41)	0.314	–		1.04 (0.36−2.99)	0.941

Crude Model: Unadjusted model. Adjusted Model: adjusted for schizophrenia, sex, marriage, education, smoking habit, age, RBANS score, interaction term between schizophrenia and age, interaction term between schizophrenia and sex. RBANS: Repeatable Battery for the Assessment of Neuropsychological Status; OR: Odds ratios; CI: confidence interval.

### 3.5 Correlation between PANSS score and FI-Lab

**Fig. 2:**
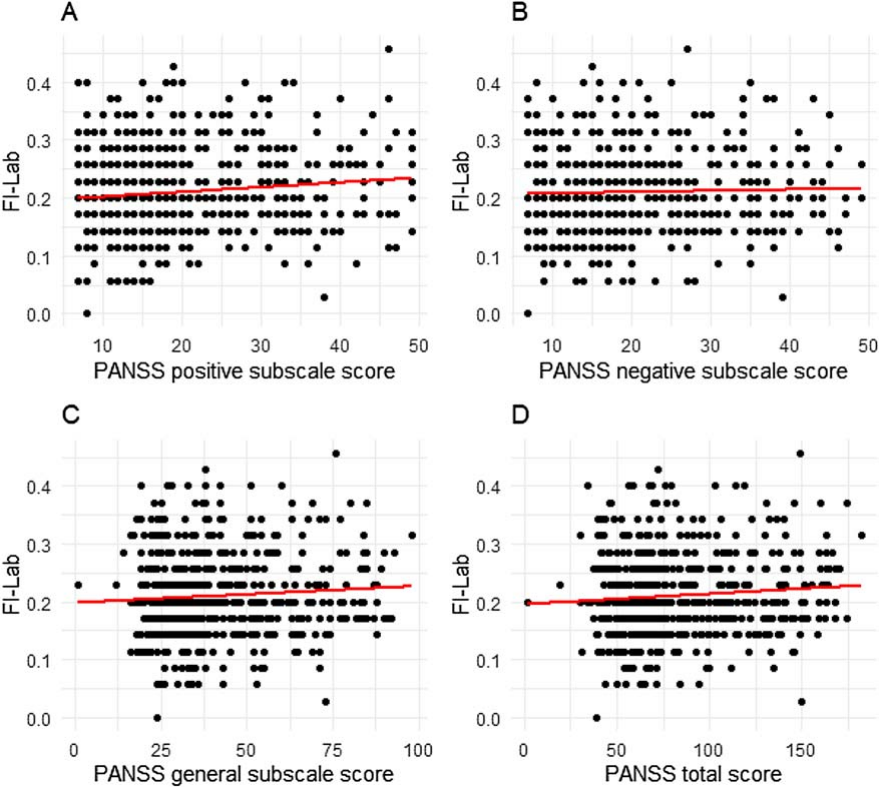
Relationship between FI-Lab and PANSS score in schizophrenia patients. Correlation analysis between FI-Lab and (A) PANSS positive subscale score; (B) PANSS negative subscale score; (C) PANSS general subscale score; (D) PANSS total score.

Additionally, the relationship between FI-Lab and schizophrenia symptoms was explored. Correlation analysis showed a positive association between FI-Lab values and the severity of schizophrenia symptoms, indicating more severe symptoms with higher level of frailty. The correlation coefficients were 0.116 (p = 0.002) for positive subscale score; 0.057 (p = 0.030) for negative subscale score; 0.096 (p = 0.010) for general subscale score; 0.086 (p = 0.019) for PANSS total score (Fig. 2).

## 4 Discussion

This study demonstrated that schizophrenia patients had higher FI-Lab across all age groups compared to the general population, indicating an elevated risk of frailty from early life stages. Additionally, a positive correlation was observed between FI-Lab and the severity of schizophrenia symptoms as measured by the PANSS scale. These findings suggest that schizophrenia may accelerate the frailty process.

Traditionally, frailty is considered to begin in older age, often overlooking the decline in physical and brain functions in patients with early-stage mental illnesses. Previous studies on older adults with schizophrenia have found that the prevalence of frailty was high in those with schizophrenia and ranged widely (10.2%–89.7%) due to the heterogeneity of study populations and different assessment of frailty [[10], [11], [12]]. The prevalence of frailty was higher than that in the general population. This trend aligns with our findings in individuals aged 60 and above. Additionally, we conducted the same analysis on younger populations and found similar results in schizophrenia patients.

Frailty phenotypes and frailty index constructed by clinical factors are often used to assess frailty in other studies. These assessment instruments focus on physical performance and self-reported health issues. FI-Lab provides a direct, quantifiable measure based on laboratory markers. This approach highlights the systemic impact of schizophrenia, which not only affects mental health but also potentially disrupts the homeostasis of the entire biological system [25]. Moreover, by including early-stage schizophrenia patients, we observed that the values of FI-Lab have already increased in young patients. This finding supports the previous hypothesis that “schizophrenia accelerates frailty”.

In the analysis of the relationship between symptom severity (measured by PANSS) and FI-Lab within the schizophrenia group, we found a relatively low correlation between psychiatric symptom severity and frailty. This suggests that frailty in schizophrenia may be influenced by factors beyond symptom severity alone, such as lifestyle, physical comorbidities, or socioeconomic status. Previous studies have indicated that the severity of symptoms in patients with schizophrenia is related to aspects like quality of life, social functioning, and daily activity capacity, all of which are also associated with frailty. Therefore, while the direct correlation between PANSS and FI-Lab may be low, it is plausible that symptom severity indirectly affects frailty through these mediating factors.

This research provides novel evidence of the connection between schizophrenia and frailty, a phenomenon driven by multiple underlying factors. Several possible mechanisms may help to explain the associations. Biologically, patients with schizophrenia exhibit significant increases in inflammatory responses and oxidative stress, both of which can lead to cellular and tissue damage, thereby accelerating the frailty process [26]. Additionally, neurobiological changes such as neuronal loss and synaptic dysfunction are associated with the brain's aging process [27]. Psychological stress, commonly present in individuals with mental illness, can perpetuate hormonal imbalances, particularly the sustained elevation of cortisol, which may disrupt intracellular repair mechanisms and further accelerate physiological aging [28]. Accelerated frailty in schizophrenia can be a part of the general pathology of this illness or caused by external factors, including unhealthy habits (smoking and lack of physical exercise), substance and medication use [29]. The significant laboratory differences observed between schizophrenia patients and controls in this study suggest distinct physiological and metabolic alterations associated with schizophrenia (Table S2). Elevated hematological markers indicate a potential inflammatory or immune response, while increased glucose metabolism markers imply a higher risk of metabolic disorders. Furthermore, dysregulated lipid profiles highlight broader metabolic imbalances in this population. These findings emphasize the importance of comprehensive metabolic monitoring and targeted interventions to effectively manage the comorbidities commonly associated with schizophrenia.

However, the cross-sectional design of this study limits our ability to infer causal relationships. Future research should be conducted in a longitudinal cohort to observe the dynamic relationship between schizophrenia and frailty. Additionally, frailer individuals tend to be biologically older, prompting the question of whether schizophrenia may accelerate the aging process. This hypothesis must still be tested in future studies using biomarkers of the aging, such as epigenetic clocks, inflammatory markers, or senescence-associated secretory phenotype (SASP).

## 5 Conclusion

This study provides new evidence of the relationship between schizophrenia and frailty using a laboratory-based frailty index. The findings suggest that patients with schizophrenia may be at an increased risk of frailty. This insight emphasizes the need for early intervention strategies in treating schizophrenia to improve the overall health of patients and delay the progression of frailty.

## CRediT authorship contribution statement

**Shun Yao:** Data curation, Conceptualization, Methodology, Software, Visualization, Validation, Writing – original draft; Writing – review & editing, Funding acquisition; **Lijun Wang:** Data curation, Investigation. **Zhiying Yang:** Investigation, Methodology. **Yichong Xu:** Investigation, Methodology. **Xiaoqing Zhang:** Investigation, Methodology. **Yuan Shi:** Data curation, Project administration. **Donghong Cui:** Conceptualization, Data curation, Validation, Funding acquisition, Methodology, Project administration, Resources, Supervision, Writing – original draft, Writing – review & editing. All authors approved the final version for publication.

## Funding

This research was supported by grants from the National Key Research and Development Program of China (No: 2017YFC0909200), and the National Natural Science Foundation of China (No: 82271544) for Dong Hong Cui; the grant from Shanghai Mental Health Center (2021-YJ09) and the grant from Shanghai Mental Health Center (2021-QH-02) for Shun Yao.

## Declaration of competing interest

The authors declare no conflicts of interest.

## References

[1] Clegg A., Young J., Iliffe S., Rikkert M.O., Rockwood K. (2013). Frailty in elderly people. Lancet.

[2] Morley J.E., Vellas B., van Kan G.A., Anker S.D., Bauer J.M., Bernabei R. (2013). Frailty consensus: a call to action. J Am Med Dir Assoc.

[3] Jauhar S., Johnstone M., McKenna P.J. (2022). Schizophrenia. Lancet.

[4] Correll C.U., Solmi M., Croatto G., Schneider L.K., Rohani-Montez S.C., Fairley L. (2022). Mortality in people with schizophrenia: a systematic review and meta-analysis of relative risk and aggravating or attenuating factors. World Psychiatry.

[5] Yan H., Huang Z., Lu Y., Qiu Y., Li M., Li J. (2023). Associations between metabolic disorders and sleep disturbance in patients with schizophrenia. Compr Psychiatr.

[6] Suetani S., Honarparvar F., Siskind D., Hindley G., Veronese N., Vancampfort D. (2021). Increased rates of respiratory disease in schizophrenia: a systematic review and meta-analysis including 619,214 individuals with schizophrenia and 52,159,551 controls. Schizophr Res.

[7] Nielsen R.E., Banner J., Jensen S.E. (2021). Cardiovascular disease in patients with severe mental illness. Nat Rev Cardiol.

[8] Adamowicz D.H., Lee E.E. (2023). Dementia among older people with schizophrenia: an update on recent studies. Curr Opin Psychiatr.

[9] Kirkpatrick B., Messias E., Harvey P.D., Fernandez-Egea E., Bowie C.R. (2008). Is schizophrenia a syndrome of accelerated aging?. Schizophr Bul.

[10] Pearson E., Siskind D., Hubbard R.E., Gordon E.H., Coulson E.J., Warren N. (2022). Frailty and severe mental illness: a systematic review and narrative synthesis. J Psychiatr Res.

[11] Aprahamian I., Landowski A., Ahn F.O., Neves B.A., Rocha J.T., Strauss J. (2022). Frailty in geriatric psychiatry inpatients: a retrospective cohort study. Int Psychogeriatr.

[12] Tsai H.C., Wu L.H., Chang S.F. (2023). Prediction of physiological state, cognition, sensory function, and biomarkers for frailty in patients aged 55 years or more with schizophrenia. Nurs Open.

[13] Searle S.D., Mitnitski A., Gahbauer E.A., Gill T.M., Rockwood K. (2008). A standard procedure for creating a frailty index. BMC Geriatr.

[14] Mitnitski A.B., Mogilner A.J., Rockwood K. (2001). Accumulation of deficits as a proxy measure of aging. Sci World J.

[15] Howlett S.E., Rutenberg A.D., Rockwood K. (2021). The degree of frailty as a translational measure of health in aging. Nat Aging.

[16] Bersani F.S., Canevelli M., Cesari M., Maggioni E., Pasquini M., Wolkowitz O.M. (2020). Frailty Index as a clinical measure of biological age in psychiatry. J Affect Disord.

[17] Ma T., Cai J., Zhu Y.S., Chu X.F., Wang Y., Shi G.P. (2018). Association between a frailty index based on common laboratory tests and QTc prolongation in older adults: the Rugao Longevity and Ageing Study. Clin Interv Aging.

[18] Sapp D.G., Cormier B.M., Rockwood K., Howlett S.E., Heinze S.S. (2023). The frailty index based on laboratory test data as a tool to investigate the impact of frailty on health outcomes: a systematic review and meta-analysis. Age Ageing.

[19] Blodgett J.M., Theou O., Howlett S.E., Wu F.C., Rockwood K. (2016). A frailty index based on laboratory deficits in community-dwelling men predicted their risk of adverse health outcomes. Age Ageing.

[20] Rockwood K., Blodgett J.M., Theou O., Sun M.H., Feridooni H.A., Mitnitski A. (2017). A frailty index based on deficit accumulation quantifies mortality risk in humans and in mice. Sci Rep.

[21] Malmstrom T.K., Miller D.K., Morley J.E. (2014). A comparison of four frailty models. J Am Geriatr Soc.

[22] Rockwood K., Andrew M., Mitnitski A. (2007). A comparison of two approaches to measuring frailty in elderly people. J Gerontol A Biol Sci Med Sci.

[23] Kay S.R., Fiszbein A., Opler L.A. (1987). The positive and negative syndrome scale (PANSS) for schizophrenia. Schizophr Bull.

[24] Randolph C., Tierney M.C., Mohr E., Chase T.N. (1998). The repeatable Battery for the assessment of neuropsychological Status (RBANS): preliminary clinical validity. J Clin Exp Neuropsychol.

[25] Ysea-Hill O., Gomez C.J., Mansour N., Wahab K., Hoang M., Labrada M. (2022). The association of a frailty index from laboratory tests and vital signs with clinical outcomes in hospitalized older adults. J Am Geriatr Soc.

[26] Jomova K., Raptova R., Alomar S.Y., Alwasel S.H., Nepovimova E., Kuca K. (2023). Reactive oxygen species, toxicity, oxidative stress, and antioxidants: chronic diseases and aging. Arch Toxicol.

[27] Kathuria A., Lopez-Lengowski K., Watmuff B., Karmacharya R. (2023). Morphological and transcriptomic analyses of stem cell-derived cortical neurons reveal mechanisms underlying synaptic dysfunction in schizophrenia. Genome Med.

[28] Tas C., Brown E.C., Eskikurt G., Irmak S., Aydın O., Esen-Danaci A. (2018). Cortisol response to stress in schizophrenia: associations with oxytocin, social support and social functioning. Psychiatry Res.

[29] Petrican R., Fornito A., Boyland E. (2024). Lifestyle factors counteract the neurodevelopmental impact of genetic risk for accelerated brain aging in adolescence. Biol Psychiatry.

