# Supplementary material for: Accelerated pace of frailty in patients with schizophrenia
Source: J Nutr Health Aging. 2024 Nov 29;29(1):100412. doi: 10.1016/j.jnha.2024.100412 (PMC12180046; doi:10.1016/j.jnha.2024.100412)
Supplement: Supplementary file 1 [file mmc1.docx]

**Supplemental Table 1 Laboratory data used to construct the FI-Lab**

| **Standard laboratory parameters** | **Normal range** |
| --- | --- |
| Red blood cells (number/L) | 4.3×10^12^-5.8×10^12^ |
| White blood cells (number/L) | 3.5×10^9^-9.5×10^9^ |
| Neutrophils (number/L) | 1.8×10^9^-6.3×10^9^ |
| Lymphocytes (number/L) | 1.1×10^9^-3.2×10^9^ |
| Hemoglobin (g/L) | 130-175 |
| Platelets (number/L) | 125×10^9^-350×10^9^ |
| Fasting glucose (mmol/L) | 3.9-5.9 |
| Total protein (g/L) | 65.0-85.0 |
| Albumin (g/L) | 40.0-55.0 |
| Glycosylated hemoglobin (%) | 4-5.6 |
| Insulin (mIU/ml) | 5-20 |
| C-reactive protein (mg/L) | <5 |
| Urea nitrogen (mmol/L) | 3.3-8.2 |
| Uric acid (μmol/L) | 240.0-490.0 |
| Creatinine (μmol/L) | 53.0-140.0 |
| Total cholesterol(mmol/L) | 2.8-5.7 |
| HDL-C (mmol/L) | 1.16-1.42 |
| LDL-C (mmol/L) | <2.59 |
| Triglyceride (mmol/L) | <1.7 |
| Apolipoprotein A1 (g/L) | 1.1-1.72 |
| Apolipoprotein E (g/L) | 30-50 |
| Lactate dehydrogenase (U/L) | 120-250 |
| Creatine kinase (U/L) | 18-198 |
| Creatine kinase isoenzymes (U/L) | <25 |
| ALT (IU/L) | <50 |
| AST (IU/L) | <40 |
| FT3 (pmol/L) | 2.3-4.1 |
| FT4 (pmol/L) | 10-23 |
| TSH (μIU/ml) | 0.4-4 |
| Ferritin (μg/L) | 20-200 |
| Folic acid (nmol/L) | 10.4-42.4 |
| SBP (mmHg) | 90-140 |
| DBP (mmHg) | 60-90 |
| BMI (kg/m^2^) | 18.5-28.0 |

HDL-C: High-Density Lipoprotein Cholesterol; LDL-C: Low-Density Lipoprotein Cholesterol; ALT: Alanine Aminotransferase; AST: Aspartate Aminotransferase; FT3: Free Triiodothyronine; FT4: Free Thyroxine; TSH: Thyroid-Stimulating Hormone; SBP: Systolic Blood Pressure; DBP: Diastolic Blood Pressure; BMI: Body Mass Index.

**Supplemental Table 2 Laboratory parameter differences in schizophrenia vs. control group**

| **Standard laboratory parameters** | **Schizophrenia** | | **Control** | | **χ2** | **P-value** |
| --- | --- | --- | --- | --- | --- | --- |
| Red blood cells | 155 | 25.83% | 92 | 17.76% | 10.53 | 0.001 |
| White blood cells | 189 | 31.50% | 101 | 19.50% | 20.84 | <0.001 |
| Neutrophils | 149 | 24.83% | 79 | 15.25% | 15.72 | <0.001 |
| Lymphocytes | 133 | 22.17% | 83 | 16.02% | 6.73 | 0.009 |
| Hemoglobin | 192 | 32.00% | 134 | 25.87% | 5.06 | 0.024 |
| Platelets | 87 | 14.50% | 80 | 15.44% | 0.19 | 0.659 |
| Fasting glucose | 156 | 26.00% | 83 | 16.02% | 16.46 | <0.001 |
| Total protein | 101 | 16.83% | 88 | 16.99% | 0.00 | 0.945 |
| Albumin | 144 | 24.00% | 106 | 20.46% | 2.00 | 0.157 |
| Glycosylated hemoglobin | 180 | 30.00% | 98 | 18.92% | 18.27 | <0.001 |
| Insulin | 47 | 7.83% | 42 | 8.11% | 0.03 | 0.866 |
| C-reactive protein | 193 | 32.17% | 91 | 17.57% | 31.27 | <0.001 |
| Urea nitrogen | 127 | 21.17% | 60 | 11.58% | 18.33 | <0.001 |
| Uric acid | 124 | 20.67% | 119 | 22.97% | 0.87 | 0.351 |
| Creatinine | 175 | 29.17% | 132 | 25.48% | 1.89 | 0.169 |
| Total cholesterol | 154 | 25.67% | 97 | 18.73% | 7.69 | 0.006 |
| HDL-C | 151 | 25.17% | 73 | 14.09% | 21.28 | <0.001 |
| LDL-C | 198 | 33.00% | 101 | 19.50% | 25.87 | <0.001 |
| Triglyceride | 183 | 30.50% | 91 | 17.57% | 25.13 | <0.001 |
| Apolipoprotein A1 | 155 | 25.83% | 102 | 19.69% | 5.92 | 0.015 |
| Apolipoprotein E | 118 | 19.67% | 103 | 19.88% | 0.01 | 0.927 |
| Lactate dehydrogenase | 149 | 24.83% | 96 | 18.53% | 6.45 | 0.011 |
| Creatine kinase | 94 | 15.67% | 72 | 13.90% | 0.69 | 0.407 |
| Creatine kinase isoenzymes | 159 | 26.50% | 133 | 25.68% | 0.10 | 0.754 |
| ALT | 55 | 9.17% | 50 | 9.65% | 0.08 | 0.781 |
| AST | 43 | 7.17% | 40 | 7.72% | 0.12 | 0.724 |
| FT3 | 76 | 12.67% | 65 | 12.55% | 0.00 | 0.953 |
| FT4 | 70 | 11.67% | 62 | 11.97% | 0.02 | 0.876 |
| TSH | 85 | 14.17% | 64 | 12.36% | 0.79 | 0.374 |
| Ferritin | 30 | 5.00% | 28 | 5.41% | 0.09 | 0.761 |
| Folic acid | 62 | 10.33% | 47 | 9.07% | 0.50 | 0.479 |
| SBP | 135 | 22.50% | 79 | 15.25% | 9.44 | 0.002 |
| DBP | 119 | 19.83% | 72 | 13.90% | 6.91 | 0.009 |
| BMI | 167 | 27.83% | 102 | 19.69% | 10.09 | 0.001 |

Table S2 displays the number and proportion of individuals with abnormal laboratory parameters in the schizophrenia and control groups, along with chi-square values and p-values for each parameter.
